# Cause of death certificates in nursing homes: Does quality matter? A retrospective review from two counties in Norway

**DOI:** 10.1177/14034948231187512

**Published:** 2023-07-26

**Authors:** Hanna M. Eng, Christian L. Ellingsen, Anne G. Pedersen, G. Cecilie Alfsen

**Affiliations:** 1 Department of Pathology, Akershus University Hospital, Norway; 2 Department of Pathology, Stavanger University Hospital, Norway; 3 Norwegian Cause of Death Registry, Department of Health Registries, Norwegian Institute of Public Health, Norway; 4 Faculty of Medicine, University of Oslo, Norway

**Keywords:** Death certification, cause of death, cause of death statistics, garbage code, quality, nursing home

## Abstract

**Aims:**

One half of Norwegians die in nursing homes, where death certificates (DCs) are completed by two types of physicians: in-house physicians or physicians on call. The aims of this study were to examine differences in the quality of DCs due to type of physician and to uncover possible implications of errors for the public statistics.

**Methods:**

DCs from the year 2013 from nursing homes in the catchment area of Akershus University Hospital were examined with regard to logical deficiencies, garbage code diagnoses and type of certifying physician. In one third of cases, the registered causes of death were compared to information in the medical records.

**Results:**

A total of 873 DCs from 24 nursing homes were evaluated. Physicians on call certified 46% of all deaths. Logical deficiencies were found in 34% of all DCs and were more common in DCs from physicians on call. Garbage code diagnoses were used in every third DC, with ‘sudden death’ or ‘cause of death unknown’ preferred by physicians on call and ‘unspecified pneumonia’ preferred by in-house physicians. Comparisons against medical records uncovered missing information in 49% and 35% of DCs from physicians on call and in-house physicians, respectively. A dementia diagnosis was frequently overlooked by both physician types. Garbage code diagnoses were more common in DCs with missing information from medical records.

**Conclusions:**

**Error rates in DCs in nursing homes in Norway are high. The results raise concerns about the validity of public cause of death statistics.**

## Introduction

Populations are ageing in developed countries [1], including Norway [2], where one half die in nursing homes. As cause of death statistics are tools for politicians and health administrators to manage health care, the quality of cause of death certificates (DCs) from the elderly is of increasing importance.

Cause of death statistics are mainly based on DCs, which are completed by physicians. In Norway, no research has yet evaluated the impact of the different types of physicians completing the DCs on the quality of the DCs. A significant proportion of DCs from nursing homes are not completed by the in-house nursing home physicians but rather by physicians on call from the local emergency centre. Thus, these DCs are suitable for evaluating the effect of possible differences due to physician type.

Elderly people often suffer from multiple diseases, and determination of cause of death in this group is challenging. It is well known that the quality of cause of death statistics is reduced in higher age groups [3,4]. The need to uncover factors contributing to reduced quality is urgent, as more precise statistics are required to allow for necessary adaptions of health and care services in response to changing age profiles.

The information on DCs should be completed according to guidelines from the World Health Organization (WHO) [5]. Part I of the DC contains the chain of events from the underlying cause of death, defined as the illness or injury that started the morbid conditions leading directly to death. The order of the chain of events should be logical, in accordance with medical knowledge of disease development. The physicians are also required to choose only one underlying condition – a consideration which may be especially problematic in patients with multiple diseases or long clinical histories.

Contributing causes are placed in part II of the DC and are defined as other significant conditions that may have contributed to the death, but without being directly or causally related to the condition that caused the death. An example would be a patient with chronic obstructive pulmonary disease (COPD) dying after an infection with the SARS-CoV-2 virus. The underlying illness causing death would be the viral infection, which should be placed in part I, while COPD would be registered as a contributing illness in part II. It is the underlying illness or injury in part I of the DC that is most relevant to the cause of death statistics.

After being completed by the physician, the DC is forwarded to the Norwegian Cause of Death Registry (NCoDR). The NCoDR processes the diagnoses into codes from the International Statistical Classification of Diseases and Related Health Problems, presently the 10th revision (ICD-10) [5], using an electronic system for automated coding [6]. Included in the ICD classification are rules for selection of underlying cause of death in cases of logical errors or imprecise diagnoses. Thus, due to adjustments in accordance with international guidelines, the final cause of death statistics may differ from the original diagnoses by the physicians on the DCs.

Many diagnoses, for example ‘multi-organ failure’, have little information value regarding the underlying cause of death. The Global Burden of Disease study (GBD) [7] uses the term ‘garbage codes’ for ICD codes that do not provide sufficient information about the underlying cause of death, grading these based on information value [8,9]. The proportion of garbage codes is used as one measure of the quality of cause of death statistics [10].

### Aims

This study aimed to investigate differences in quality in DCs from the two different types of physicians completing DCs in nursing homes (in-house physicians and physicians on call), with respect to (a) the prevalence of logical deficiencies, (b) the prevalence of garbage code diagnoses and (c) discrepancies between information on DCs and the medical records.

## Methods

### Materials

Deaths in 2013 in nursing homes from the catchment area of Akershus University Hospital, including parts of the neighbouring counties Akershus and Oslo, were evaluated. Only nursing homes with in-house physicians in ⩾50% of positions were selected. Final ICD-10 codes for underlying cause of death and copies of original DCs were received from the NCoDR, and sex, age, entered conditions and the type of certifying physician were registered. The type of physician was classified as in-house nursing home physician (‘in-house physician’) or physician on call from the local emergency team (‘physician on call’).

Logical deficiencies in the chain of events in DCs were assessed by two pathologists with expertise in cause of death certification (H.M.E. and C.A.). Disagreements were discussed, and consensus was achieved in all cases. Logical deficiencies were categorised as type A: errors in the sequence of chain of events in part I; type B: underlying cause of death placed in part II; type C: multiple independent causes of death in part I; or type D: cause of death given both as underlying (in part I) and contributing cause of death (in part II).

Based on the information from DCs, all cases were assigned a disease category in accordance with the ICD-10 or, if several underlying causes given, grouped as ‘classification not possible’.

Diagnoses subject to garbage codes (‘garbage code diagnoses’) were classified according to definitions from the GBD [7,8] and grouped as major (diagnoses with serious/substantial policy implications) or minor (diagnoses with important/limited policy implications).

Medical records were electronically available from five out of six nursing homes in Oslo. The records were reviewed and compared with the original DC and the final ICD-10 code for underlying cause of death. Discrepancies were registered as either change in ICD-10 chapter or block within chapter.

A chi-square test (IBM SPSS Statistics for Windows v27; IBM Corp., Armonk, NY) was used to estimate differences in proportions. *p*-Values <0.05 were considered statistically significant.

### Ethical approval

The study was approved by the Norwegian Regional Committee for Medical and Health Research Ethics (2015/627), the Norwegian Data Protect-ion Authority (16/01543-1/GRA) and the Data Protection Officer at Akershus University Hospital (15-121).

## Results

### Nursing homes and type of physician

A total of 1026 deaths were reported from 18 nursing homes in Akershus and six in Oslo in 2013. DCs from 873 deaths were examined, after excluding DCs completed in hospitals (*n*=82), after forensic examinations (*n*=6) and duplicates and missing or unreadable certificates (*n*=65).

The majority of DCs were from the more rural county of Akershus, with on average fewer deaths per nursing home than in the city county Oslo and with more extensive use of physicians on call for certifying deaths (56% in Akershus vs. 29% in Oslo; Table I). Overall, the proportion of DCs from physicians on call amounted to 46% (399/873).

**Table I.** Cause of death certificates from nursing homes in Akershus and Oslo counties in Norway in 2013, according to age, gender and type of completing physician (*N*=873).

	All	Akershus	Oslo
Localisation of nursing home, *n*	24	18	6
DCs, *n*	873	549	324
DCs per nursing home, median (range)	36 (9–90)	31 (9–46)	54 (24–90)
Age (years), median (range)			
Females	88 (17–104)	87 (17–104)	89 (46–102)
Males	84 (45–102)	84 (45–100)	83 (53–102)
Females, *n* (%)	528 (60)	327 (60)	201 (62)
DCs completed by physician on call, *n* (%)	399 (46)	306 (56)	93 (29)

DC: death certificate.

### Types of errors

Logical deficiencies were found in 34% (298/873) of all DCs (Table II). Deficiencies were seen more often in DCs from physicians on call than from in-house physicians (39% and 30%, respectively; *p*=0.003).

**Table II.** Underlying cause of death in DCs from nursing homes, classified according to the ICD-10, and the finding of logical deficiencies in the construction of underlying cause of death (*N*=873).

ICD-10 chapter	ICD-10 codes	Type of disease as underlying cause of death in DCs	Total number	DCs with logical deficiencies, *n* (%)
I	A00–B99	Infectious and parasitic diseases	21	5 (24)
II	C00–D48	Neoplasms	176	34 (19)
III	D50–D89	Diseases of the blood and blood-forming organs	5	0 (0)
IV	E00–E90	Endocrine, nutritional and metabolic diseases	21	11 (52)
V	F00–F99	Mental, behavioural and neurodevelopmental disorders	39^a^	12 (31)
VI	G00–G99	Diseases of the nervous system	25^b^	5 (20)
IX	I00–I52, I70–I99	Diseases of the circulatory system, except vessels in the brain	129	30 (23)
IX	I60–I689	Diseases of vessels in the brain	46	10 (22)
X	J00–J99	Diseases of the respiratory system	136	32 (24)
XI	K00–K93	Diseases of the digestive system	14	5 (36)
XII	L00–L99	Diseases of the skin and subcutaneous tissue	3	1 (33)
XIII	M00–M99	Diseases of the musculoskeletal system and connective tissue	3	2 (67)
XIV	N00–N99	Diseases of the genitourinary system	34	13 (38)
XVIII	R00–R99	Symptoms, signs and abnormal clinical and laboratory findings, not elsewhere classified	70	12 (17)
XIX	S00–T98	Injury, poisoning and certain other consequences of external causes	4	2 (50)
XX	V0n–Y9	External causes of morbidity and mortality	31	8 (26)
Classification not possible^c^			116	116 (100)
In total			873	298 (34)

a Including 36 cases of dementia (unspecified type 33, vascular type 3).

b Including 19 cases of dementia (Alzheimer’s 10, Parkinson’s disease 8, alcohol related 1).

c Several underlying causes of death in part I (103 DCs) or in part II (13 DCs).

ICD-10: International Statistical Classification of Diseases and Related Health Problems, 10th revision.

The listing of several independent causes of death (type C errors) was significantly more common in DCs completed by physicians on call, with 16% (65/399) of DCs from physicians on call listing several independent diagnoses in part I compared with 8% (38/474) of DCs from in-house physicians (*p*<0.001). No significant differences were found for other types of error. Type A, type B and type C errors were found in 14%, 8% and 1% of DCs from in-house physicians and in 12%, 7% and 1% from physicians on call, respectively.

No significant differences in error type rates were seen between the physician types with regard to geographical localisation of the nursing homes.

DCs with no distinct underlying cause of death, with symptom diagnoses (R00–R99) or with no possible classification accounted for 21% of all cases (186/873; Table II). The majority of DCs with no possible classification listed several independent underlying causes of death in part I (type C errors: 103/116 cases).

Female deaths were significantly more often given a symptom diagnosis: 83% of symptom diagnoses (R00–R99) were in women (58/70; *p*=0.00006). No other significant sex differences were seen, whether in the use of ICD chapters, unclassifiable DCs or proportions of logical deficiencies.

Patients dying of neoplasms (C00–D48) or injuries/poisonings (S00–T98) were on average younger, with mean ages of 76.5 years and 69.5 years, respectively.

### Garbage code diagnoses

Garbage code diagnoses were used in every third DC (298/873; Table III), with major and minor types constituting 18% and 16% (161 and 137 cases), respectively, of all cases. Overall, garbage code diagnoses were used equally as often by in-house and physicians on call (35% and 34%, respectively), albeit with significant differences in the use of diagnoses. ‘Cause of death unknown’ (R96) was significantly more common from physicians on call (*p*=0.011), while ‘unspecified pneumonia’ (J189) was used significantly more often by in-house physicians (*p*=0.004).

**Table III.** Garbage code diagnoses as underlying cause of death in DCs from nursing homes, according to type of certifying physician (*N*=298).

Garbage code, ICD-10 [11]	Garbage code diagnoses in DC	Total number	Certified by physician on call
*Major*			
R96	Sudden death, cause of death unknown	29	20
I50.9	Heart failure, unspecified	27	17
X59	Exposure to unspecified factor	26	12
R54	Senility, including high age	22	10
I10	Essential (primary) hypertension	11	7
R63	Nutritional disorder, including anorexia	10	4
N19	Kidney failure, unspecified	7	4
E86	Dehydration	5	3
J81	Pulmonary oedema	4	1
A49.9	Bacterial infection, unspecified	4	2
I26	Pulmonary embolism	3	2
I46.9	Cardiac arrest, unspecified	3	1
A41.9	Septicaemia, unspecified	2	0
R64	Cachexia	2	0
I50.1	Left ventricle failure, including cardiogenic pulmonary oedema	2	1
I50	Heart failure	1	0
K72.9	Hepatic failure, unspecified	1	1
R68.8	Multi-organ failure	1	1
R09.2	Respiratory arrest	1	1
*Minor*			
J18.9	Pneumonia, unspecified	84	28
I64	Stroke, unspecified	28	11
E14	Diabetes mellitus, unspecified	7	6
C80.0, C80.9	Malignant neoplasm, primary site unknown or unspecified	6	3
I51.6	Cardiovascular disease, unspecified	5	0
I69.4	Sequelae of stroke, unspecified	4	2
I49.9	Cardiac arrhythmia, unspecified	2	1
J18.0	Pneumonia, organism unspecified	1	1
	**Total**	298	139

With the exception of garbage code group X59 (‘exposure to unspecified factor’), female sex dominated within major garbage code groups. Seventy-three per cent of all major garbage codes were allocated to women (117/161; *p*=0.0005), who in average were older than women with no major garbage codes (mean ages of 91 and 84 years, respectively). No significant differences in sex or age distributions were seen within the minor garbage code groups.

Overall, no significant geographical differences in use of garbage code diagnoses were found (Akershus 177/549 (32%), Oslo 121/324 (37%); *p*=0.124).

### Comparison to medical records

A review of the medical records identified different causes of death from the ones registered as final diagnoses in 39% (106/274) of cases (Table IV). Important information was missing more often in DCs from physicians on call (36/73; *p*=0.029). However, in-house physicians also ignored information about underlying conditions in a third of cases (70/201). A third of examined records (32/106) contained new information implying death due to various types of dementia. The majority were females (18 females vs. 14 males) and significantly older (mean age: females 91 years vs. males 86 years). Dementia was overlooked by both physician types (in-house physicians: 20 cases; physicians on call: 12 cases). The use of garbage code diagnoses was significantly more common in DCs lacking information (31%; 33/106) than in DCs with no new findings (15%; 25/168; *p*=0.001). The level of garbage code diagnoses was highest in patients whose dementia was overlooked (44%; 14/32). The most common diagnoses in overlooked dementia cases were unspecified pneumonia (I18.9, *n*=6) and cause of death unknown (R96, *n*=4).

**Table IV.** Differences in underlying cause of death according to information from medical records in nursing homes and final ICD-10 code according to type of physician and the use of garbage code diagnoses in the DCs (*N*=274).

Difference between medical record and final ICD-10 code	Total number (%)	In-house physician	Physician on call
		*N*=201	***N*=73**
		Total number/with garbage code diagnoses	Total number/with garbage code diagnoses
No difference	168 (61)	131/20	37/5
Difference, all	106 (39)	70/22	36/11
Thereof:			
With different ICD-10 chapter	90^a^	60/19	30/9
With different ICD-10 block	16	10/2	6/2

a Including 32 deaths due to dementia (unspecified type: 28; Alzheimer’s: 2; Parkinson’s disease: 2), 13 deaths due to accidental falls or traffic accidents, seven deaths due to malignant neoplasms and five deaths due to stroke.

## Discussion

We investigated the quality of DCs from nursing homes in two counties in Norway. Logical deficiencies, use of diagnoses with low information value and a lack of important information were identified in at least a third of examined DCs. Errors by both physician groups were seen, although physicians on call more often listed several underlying causes of death, used the term ‘cause of death unknown’ more frequently and were more often ignorant of information in medical records. Death by dementia was the most commonly overlooked diagnosis from medical records.

### Strength and limitations

The material in this study is a decade old. The results, however, remain valid. From 2022, online certification of death has replaced the manual completion of DCs in Norway [12]. The online DC is structured identically, with the chain of events leading to underlying cause of death in part I and contributing diseases in part II. The online system facilitates selection of codes from the ICD-10 but has maintained the alternative option of manual entry, including entry of several diagnoses, and the possibility of sequencing errors. Knowledge of errors in the manually completed DCs may thus contribute to improvements in the online certification version.

Medical records were examined several years after death. The status of the records at the time of death certification and whether certifying physicians had access to relevant medical information are unknown. Retrospective studies may also suffer from a lack of information conveyed orally or due to undocumented circumstances. However, as the impact of missing information was compared to final cause of death data, after any corrections from physicians or the NcoDR, the proportion of DCs with missing information is considered a minimum.

It may be considered a limitation that medical records and DCs were reviewed by pathologists and not by clinicians closer to the patients. However, pathologists performing autopsies on a regular basis are trained in reading and extracting the relevant information from clinical records. Having distance from the most acute events also may allow for more objective assessments of the clinical stories.

Differences between the two counties in the use of physicians on call for cause of death certification were striking (Table 1) and may at least partially be explained by the differences in sizes of the nursing homes. The differences in numbers have been accounted for in the statistical analyses. The sizes of the nursing homes in the two counties differed due to differences in population density of rural and city areas. That only medical records from the on average larger nursing homes in Oslo were examined is a weakness of the study and means that caution is needed with regard to generalisation of the results.

### Discussion of results

Studies have assessed the importance of physician type for the quality of DCs. Younger age of certifying physicians [13] and work in non-university hospitals or nursing homes [14,15] have been linked to lower-quality DCs. Studies on DC completion by general practitioners have shown both worse [16] and better [17] quality. The importance of experience is also uncertain. One study showed worse results by senior physicians [18]. A third of hospital-employed physicians in a US study did not trust the correctness of their own or others’ entries [19]. A Norwegian study indicated that hospital-employed physicians certified too few deaths to acquire sufficient knowledge [20]. Lack of training was also discussed as a contributing factor for the more common use of garbage codes in deaths outside hospitals in Norway [4]. Nursing homes have fewer in-house physicians and more deaths per patient than hospitals do. Thus, more experience in death certifications should be expected of physicians employed in nursing home . However, apart from the level of type C errors and differences in the use of some garbage code diagnoses, differences between types of physicians in our study proved insignificant. Although physicians on call overlooked important diagnoses more frequently, the fact that in-house physicians missed important information from medical records in 35% of cases indicates that a surprisingly large proportion may not have been aware of or ignored the medical history of their patients.

The impact of missing diagnoses on the registration of dementia conditions may have serious implications for public health and care planning. The number of dementia-related deaths in Norway has increased in recent years [21]. Some of the increase is attributed to changes in cause of death coding. Our data indicate that dementia as cause of death may be seriously underestimated.

Dementia is not often communicated in DCs [22–25]. In the present study, dementia constituted 6% of causes of death according to DCs (Table II). After a review of the medical records in a third of DCs, deaths by dementia increased to 32 out of 274 cases (18%; Table IV).

Different assessment of dementia as the underlying cause of death may be due to several factors. The causal role of dementia in the chain of events leading to death may be unclear to the clinician. The timespan between casual factors may be long and a relationship thus easily overlooked. A typical example would be dementia patients who terminally suffer from malnutrition or aspiration pneumonia because of prior feeding problems. Differing opinions on the causative role of dementia conditions in general may also be a factor, especially if the patients are old and suffer from multiple illnesses. The patients with overlooked dementia conditions in the present study were on average two to three years older. However, the common use of garbage code diagnoses in this group does not indicate that the problem was choosing among several competing illnesses. A lack of understanding of the significance of information from DCs as a public management tool could also be an explanation for insufficient reading of medical records by the certifying physician.

As the NCoDR uses ICD algorithms [5] to determine the underlying cause of death, errors in the order of chain of events in part I, or placement of the underlying cause of death in part II, are often discovered and corrected. If several underlying causes of death are given, the algorithms follow the principle of choosing the first given cause based on the idea that physicians mention the most important diagnosis first. This may not always be the case, and the correction of DCs with these types of errors is fraught with uncertainty. The proportion of DCs with multiple independent causes of death (type C errors) amounted to 12% (103/873) of all cases, which is three times higher than reported in a similar study from the USA [26].

Use of imprecise diagnoses increases with age at death [27] – a fact that is confirmed by the present study. The frequent use of ‘cause of death unknown’ by physicians on call may be due to lack of information about the deceased. ‘Unspecified pneumonia’, as preferred by the in-house physicians in our study, is often a terminal complication in several underlying causes of death. An autopsy study from a geriatric hospital revealed concordance in only 10% of pneumonias [28]. The study also showed a trend not to use medical records when completing the DCs in deceased of advanced ages, but rather to choose a ‘most likely diagnosis’.

The use of diagnoses such as ‘old age’ (R54) or ‘malnutrition’ (R63) may reflect deaths without a defined fatal illness, that is, old people who simply die of age. However, garbage code diagnoses were used more frequently in DCs with overlooked medical information. A Norwegian study on DC completions in hospitals found a higher rate of major errors in DCs with imprecise diagnoses or logical deficiencies in the chain of events [20]. Thus, DCs with garbage diagnoses may indicate DCs with errors and thus serve as markers for deaths which should be investigated more closely.

## Conclusions

The proportion of errors in DCs from nursing homes in Norway is a cause for concern, not least for the estimation of deaths from dementia disorders. Surveillance of long-term effects of medical treatment and medical health prevention requires credible insight into causes of death. Targeted measures towards a more correct completion of DCs in the population of elderly, using tools from modern information technology, are necessary if data from public statistics are to be valid for future planning. Although with differences in the use of diagnoses and error rates, errors in DC completions were performed both by in-house physicians and physicians on call – indicating that knowledge of cause of death certification in nursing homes is insufficient. The recognition that physicians lack a basic understanding of the importance of cause of death certificates is important for further development of DC completion methods, not least from manual to online solutions.
